# The Neurobiological Pathogenesis of Poststroke Depression

**DOI:** 10.1155/2014/521349

**Published:** 2014-03-04

**Authors:** Chao Feng, Min Fang, Xue-Yuan Liu

**Affiliations:** ^1^The Yiwu Affiliated Hospital of Zhejiang University, School of Medicine, Huajiachi Campus, Kaixuan Road No. 268, Jianggan District, Hangzhou 310029, Zhejiang, China; ^2^Department of Neurology, Shanghai Tenth People's Hospital of Tongji University, Shanghai, China

## Abstract

Poststroke depression (PSD) is an important consequence after stroke, with negative impact on stroke outcome. The pathogenesis of PSD is complicated, with some special neurobiological mechanism, which mainly involves neuroanatomical, neuron, and biochemical factors and neurogenesis which interact in complex ways. Abundant studies suggested that large lesions in critical areas such as left frontal lobe and basal ganglia or accumulation of silent cerebral lesions might interrupt the pathways of monoamines or relevant pathways of mood control, thus leading to depression. Activation of immune system after stroke produces more cytokines which increase glutamate excitotoxicity, results in more cell deaths of critical areas and enlargement of infarctions, and, together with hypercortisolism induced by stress or inflammation after stroke which could decrease intracellular serotonin transporters, might be the key biochemical change of PSD. The interaction among cytokines, glucocorticoid, and neurotrophin results in the decrease of hippocampal neurogenesis which has been proved to be important for mood control and pharmaceutical effect of selective serotonin reuptake inhibitors and might be another promising pathway to understand the pathogenesis of PSD. In order to reduce the prevalence of PSD and improve the outcome of stroke, more relevant studies are still required to clarify the pathogenesis of PSD.

## 1. Introduction

Poststroke depression (PSD) is a common complication after stroke. There is no exact result for the prevalence of PSD. Most studies reported a prevalence of PSD from 30% to 50% and the results differed partly because of the vague definition and criteria of PSD, different observing time after stroke, and different methodologies [1]. The clinical symptoms of PSD mainly include depressed mood apathy, weight loss or gain, sleep changes, fatigue, worthlessness, and anhedonia, with the first two symptoms as the core symptoms. PSD might be associated with the impairment of other neurological functions such as learning, executive, and motor functions. Most importantly, PSD could negatively influence the functional outcome after stroke rehabilitation and is a risk factor for low quality of life after stroke and a high morbidity [2, 3].

Until now, the pathogenesis of PSD has not been well defined. Previous studies showed that PSD might be related to different factors including neurobiological, behavioral, and social factors. Among them, behavioral and social factors have been proved to be related to all kinds of depression while neurobiological changes after stroke might be more special for PSD, different from other subtypes of depression. Meanwhile, neurobiological changes after stroke are complicated with interactions among different factors; thus more studies were focused on the depression-related poststroke neurobiological changes. In this paper, the authors reviewed the possible neurobiological pathogenesis of PSD and sought to make a conclusion for the previous related studies.

## 2. Neuroanatomical Factors

### 2.1. Lesion Location Hypothesis

The hypothesis that the risk of PSD is influenced by the lesion location of stroke is most widely publicized and is meanwhile controversial among various hypotheses of the mechanisms of PSD. This hypothesis was first proposed more than 30 years ago by Robinson et al. who reported associations between laterality of experimentally induced cerebral infarction, catecholamine concentrations, and the behavior of rats [4]. Later, they further reported that strokes of left hemisphere, especially in left frontal region, were significantly associated with the depressive disorders in human beings [5]. Since then, a lot of related studies were carried out; however, the results of these studies varied a lot.

Studies in favor of the lesion location hypothesis mainly identified left hemisphere, especially left frontal lobe, and basal ganglia as the critical areas of PSD. Some researchers concluded them as “frontal subcortical circuits (FSC) [6]” and “limbic-cortical-striatal-pallidal-thalamic circuits (LCSPTC) [7]” which were also proved to be the key network that putatively modulates emotional behavior in nonstroke subjects [8]. These two circuits are similar to some extent and both consist of the frontal lobe, basal ganglia, and other adjacent structures and were supported by various studies. Tang et al. [9] studied 591 patients and found that the presence of infarcts in FSC was significantly associated with PSD (*P* = 0.03). Vataja et al. [10] also demonstrated a strong correlation between PSD and infarcts that affected FSC, especially internal capsule on the left side (OR = 3.2) and pallidum of any side (OR = 1.6). Terroni et al. [11] showed that infarctions in LCSPTC, especially in the areas of the left medial prefrontal cortex, were associated with PSD (*P* = 0.004). Some results from animal experiments were also in favor of this hypothesis. In a recent study of rats, Kronenberg et al. [12] reported that left, but not right, middle cerebral artery occlusions lead to chronic “depression-like” behavior.

The “left frontal lobe theory” and the left FSC & LCSPTC theory were dominant in the group of researchers in favor of the lesion location hypothesis. However, there were different opinions about the specific locations that were associated with PSD. For example, Dam et al. [13] and MacHale et al. [14] both showed that depression was more significantly associated with lesions involving the right cerebral hemisphere in their respective studies. In a recent MRI-based cohort study of 163 Chinese patients of stroke [15], the results showed that PSD patients had higher rates of infarcts in frontal and temporal lobes as well as in internal capsule, but no side-related association was found.

Meanwhile, some researchers were not in favor of this lesion location hypothesis at all. In the studies carried out by Caeiro et al. [16] and Aben et al. [17], the results of both proved no association between PSD and lesion location. Another prospective study of 420 patients showed no association between PSD and lesion side or location [18].

Apparently, the evidence on the lesion location hypothesis of PSD was conflicting. Some researchers analyzed the various studies in this field systematically; however their conclusions turned to be conflicting too. Carson et al. [19] searched the 95 studies about PSD and lesion location for a systematic review. The results showed that the pooled relative risk of depression after a left-hemisphere stroke compared with a right-hemisphere stroke was 0.95 (95% CI 0.83–1.10) and offered no support for the hypothesis that the risk of PSD is affected by left- or right-hemisphere stroke. In a meta-analysis study [20] which included 52 studies involving 3668 patients, the results suggested a weak relationship between PSD and right hemisphere lesion. In another systematic review which included 26 studies [21], the results suggested that left hemisphere lesion location was associated with PSD in hospital patients (OR = 1.36, 95% CI 1.05–1.76), while the right hemisphere lesion location was associated with PSD in community (OR = 0.60, 95% CI 0.39–0.92). Apparently, more studies were still needed to clarify the relation between lesion location and PSD. Besides, in order to decrease the discrepancy among studies, more work shall be needed to uniform the definition and evaluation methodology of PSD.

### 2.2. Infarction Size

Beside lesion location, the infarction size is another lesion characteristic that might be associated with the presence and severity of PSD. Large infarction might result in severe damage to the critical areas that are associated with the modulating of emotional behavior and biochemical change. The severe neurological deficit caused by large infarction could be an important social-psychological factor associated with the pathogenesis of PSD. Several studies evaluated the relation between infarction size and PSD. Vataja et al. [22] showed that lesions in FSC were larger in patients with PSD in a study of 70 patients with single lesion than other stroke patients. In a prospective study of 126 patients, Nys et al. [23] found that early PSD was significantly associated with lesion size (*P* = 0.008). In a Chinese cohort study of PSD [15], the results also suggested that the volume of acute infarcts was higher in PSD group than control group (*P* = 0.029). Unlike the controversial evidence of lesion location hypothesis, the results about the associations between PSD and infarction size seemed to be unanimous in favor of the conclusion that large infarction size was associated with PSD.

### 2.3. Vascular Depression Hypothesis

In the 1990s, researchers observed that white matter hyperintensities and silent cerebral infarctions were associated with a higher rate and greater severity of late-life-onset depression [24–26]. Based on this finding, Krishnan et al. [27] and Alexopoulos et al.[28] proposed the hypothesis of “vascular depression” which emphasized the role of cerebrovascular disease especially small vessel disease in the pathogenesis of late-life depression [29, 30] and which is supported by abundant studies [31–35]. According to this hypothesis, silent lesions disrupt the cortico-striato-pallido-thalamo-cortical pathways and then result in depressive symptoms [36], similar to the lesion location hypothesis of PSD. Cerebral lesions and the disruption of critical areas were highlighted again in the pathogenesis of depression by this hypothesis.

Vascular depression and PSD were used to be regarded as different types of depression caused by silent lesions and acute infarctions, respectively. However, some studies proved that vascular depression and PSD were not that different. Brodaty et al. [37] found that PSD was associated with the accumulation of vascular brain pathology rather than severity of single strokes. Kim et al. [38] studied 133 stroke patients and proved that the severity of deep white matter hyperintensities was related to delayed depression. Santos et al. [39] performed a detailed analysis of all types of vascular lesions and lacunes in 41 consecutively autopsied stroke cases and found that macroinfarct site was not related to the occurrence of PSD for any of the locations studied. On the contrary, higher lacune scores in basal ganglia, thalamus, and deep white matter were associated with an increased PSD risk (all *P* < 0.05). These studies suggested that the depressive symptoms were caused by the accumulation of cerebral lesions or lesions in critical areas, rather than the attack of stroke. The lesions of corresponding acute infarctions might function just like other silent lesions in the pathogenesis of PSD if they were located in some critical areas according to the lesion location hypothesis of vascular depression and PSD. Several epidemiological studies also proved evidence in favor of the “vascular depression” related hypothesis in PSD. Tennen et al. [40] recruited 102 patients within 4 months after stroke and found that hypertension was associated with poststroke depressive symptoms (*P* = 0.029). In a cohort study of 149 stroke patients, Pascoe et al. [41] observed that the level of homocysteine which was a risk factor for vascular disease was significantly associated with the presence of PSD (*P* < 0.001).

Based on the studies mentioned above, most researchers began to accept the concept of “vascular depression” as one of the potential explanations for the pathogenesis of PSD and conclude PSD into the category of vascular depression [7, 42]. However, there were also different opinions. Some researchers argued that the direct consequences of stroke itself were strongly depressogenic and could overshadow the preexisting small vascular lesions [17], and this viewpoint could be supported by several studies. In a large clinical study which included 670 geriatric patients, Mast et al. [43] observed a significant increase in the frequency of depression in patients without stroke as the burden of cerebrovascular risk factors increased; however, this effect was not observed among stroke patients. The results suggested that the burden of vascular risk factors might not affect the frequency of PSD. Similar negative results were also observed by Aben et al. [17] and Vataja et al. [10] in their respective studies.

## 3. Neuronal Biochemical Factors

### 3.1. Neurotransmitter Hypothesis

Since the emotional behaviors are regulated by different neurotransmitters especially monoamines, the dysfunction of monoamines due to different conditions could result in different kinds of psychiatric symptoms including depression [44]. This amine hypothesis could be conceived as the supplement or further explanation of lesion location hypothesis when it is used to explain the pathogenesis of PSD or vascular depression [28]. According to this hypothesis, cerebral lesions interrupt the projections ascending from midbrain and brainstem, passing through thalamus and basal ganglion, and reaching the frontal cortex and then result in the decrease or decreased bioavailability of biogenic amines, including serotonin (5-HT), dopamine (DA), and norepinephrine (NE), thus resulting in depressive symptoms [2]. This hypothesis was first described by Robinson and Bloom in the 1970s after they observed the association between the decrease of catecholamine concentrations and abnormal activity of rats [45], and it was supported by different kinds of studies involving amines, receptors, and mRNA. Gao et al. [46] measured the concentrations of plasma and CSF serotonin in 60 patients and observed a significant reduction of both serum and CSF serotonin (both *P* < 0.01) in PSD patients. Winter et al. [47] found that lesions of dopaminergic neurons in the substantia nigra pars compacta and in the ventral tegmental area enhance depressive-like behavior in rats. Wang et al. [48] investigated the 5-HT (1A) receptor and mRNA level in the hippocampus of rats and observed a significantly decreased protein of 5-HT (1A) receptors and mRNA level in PSD models compared to other rats. Now drugs involved in the amino-neurotransmitters including SSRIs and SNRIs which could decrease the reuptake of serotonin are widely used for the studies and treatment of depression including PSD. Their effect also provided evidence in favor of the amine hypothesis in the pathogenesis of PSD [12, 49, 50].

Glutamate is another neurotransmitter that might be involved in the pathogenesis of depression [51]. Glutamate is abundant in the brain and functions as an excitatory neurotransmitter normally through the activation of NMDA receptors [52]. However, an overload of glutamate might result in deleterious effect. Some researchers proposed that the disruption in glutamatergic neurotransmission might be a possible etiology of clinically observed depression [51, 53], and now NMDA receptor has become a new target of antidepressant drugs [54]. Recently, several studies proved that glutamate might also be involved in the pathogenesis of PSD. Glodzik-Sobanska et al. [55] assessed the biochemical profiles in prefrontal regions of 26 stroke patients with proton magnetic resonance spectroscopy (MRS) and found that patients with stroke had higher glutamate+ glutamine/Cr ratio. Another MRS study [56] also showed that PSD patients had a higher glutamate level than other stroke patients without depression. However, it is still unclear whether and how the high glutamate level is associated with stroke onset. Unlike monoamines which could be decreased by the disruption of critical structures of brain, the level of glutamate might not be affected by specific lesions. The metabolic and biochemical change due to stroke might be a better answer for the high glutamate level.

### 3.2. Immune Dysfunction Hypothesis

Recently, the immunological process underlying ischemia stroke has been highlighted. Abundant studies proved the association between stroke and the increase of various proinflammatory cytokines, including members of interleukin (IL), tumor necrosis factor (TNF), and interferon (IFN) families [57]. Interestingly, depression was also proved to be associated with the increased inflammatory response involving a higher level of IFN-*γ*, IL-1*β*, TNF-*α*, IL-6, and IL-1 and decreased IL-10 [58, 59]. Some researchers even suggested the concept of depression as a dysfunction of immune system [60, 61]. According to this theory, the activated inflammatory response could be a possible explanation for the high prevalence of depressive symptoms after stroke [62].

There are different opinions on the specific mechanisms of inflammation-related PSD. Tissue destruction and cell death were most believed to be the bridge between inflammation and PSD. Animal models revealed that several proinflammatory cytokines including IL-1*β* and TNF-*α* were elevated in the hippocampus and striatum which might be the critical areas of mood disorder and could increase infarction size and oedema formation [63–65], while the inhibition of these factors might reduce infarction size [66]. In clinical studies, similar effect of IL-1*β* and TNF-*α* was also demonstrated [67]. Inflammatory cytokines might also play an important role in the regulating of cell death, including apoptosis and necrosis [68], especially the cell death of vulnerable areas such as hippocampus [69]. Increased cell death results in the enlargement of cerebral infarction and might also be directly associated with depressive symptoms [70]. Several animal models of depression showed that apoptosis of brain structures such as hippocampus and amygdala was enhanced [71, 72]. Antidepressant treatment was also proved to be neuroprotective through the regulation of apoptosis [71]. Further studies about the mechanism of cytokine-induced cell death revealed that intracellular Ca2+, glutamate excitotoxicity, and free radicals might be key factors. For example, IL-1 and IL-6 might interrupt the metabolic system of glutamate and lead to enhanced neurotoxicity [73]. However, considering the complexity of cytokine network and cell death, in the future more studies are still required.

Proinflammatory cytokines might also interfere with the synthesis and metabolism of amine neurotransmitters [74]. This mechanism was supported by many studies. Miller [75] proved that administration of IFN-*α* could influence the synthesis and reuptake of serotonin. Felger et al. [76] established a nonhuman primate model of cytokine-induced depression with rhesus monkeys and observed a lower dopamine metabolite concentration in CSF. Zhu et al. [77] reported that IL-1*β* and TNF-*α* could activate serotonin transporters and thus increase the uptake of serotonin. However, there were different opinions about the specific mechanism of how inflammation interferes with serotonin. For example, Dantzer et al. [78] pointed out that the brain under inflammatory response could compensate for the decrease in circulating tryptophan which is the main source of serotonin production; thus, the increased uptake of serotonin through the activation of serotonin transporter might be a more reasonable hypothesis rather than the decreased serotoninergic neurotransmission.

### 3.3. Hypothalamic-Pituitary-Adrenal (HPA) Axis Activation Hypothesis

HPA axis functions normally as the response to environmental stressors. HPA axis activation is quite common after stroke, with elevated glucocorticoid level, that is, hypercortisolism, as the most prominent feature [79, 80]. The specific mechanisms for the activation of HPA axis after stroke might be various. Most believed that the stress from stroke attack could activate the HPA axis just like other stressors. Some other researchers speculated that cytokines might also be involved in pathogenesis of HPA hyperactivity [80]. The effect of glucocorticoid on stroke is complicated. It might improve the neurological outcome [81]; meanwhile, it might also be related to PSD. In a prospective study of 70 patients with acute stroke, Astrom et al. [82] found that high postdexamethasone cortisol levels were significantly higher in patients with late-onset major depression (*P* < 0.001), thus proving the association between hypercortisolism and PSD.

Glucocorticoid has been proved to be involved in the regulation of neural survival, the emotional appraisal of events [83]. This might be the basis of the glucocorticoid-related depression. However, the specific mechanism of hypercortisolism-related depression is still unclear. Previous studies showed that major depression was associated with glucocorticoid resistance [84], which could be induced by hypercortisolism, and resulted in glucocorticoid receptor dysfunction [85]. HPA axis dysfunction is closely associated with cytokines in the pathogenesis of PSD, although it is still unclear whether cytokines or glucocorticoids come first in PSD [86]. Several studies showed that cytokines might induce hypercortisolism and glucocorticoid resistance through the inhibition of glucocorticoid receptor [87, 88]. Vice versa, glucocorticoid could increase the levels and regulate the function of some cytokines, including IL-1*β*, IL-6, and TNF-*α*.

Hypercortisolism might also be related to monoamine dysfunction. In a recent clinical study, Reimold et al. [89] investigated the interrelation of cortisol response and thalamic serotonin transporter level with positron emission tomography, and they found that reduced thalamic serotonin transporter level was significantly associated with increased cortisol response (*P* < 0.01). Besides, the effect of glucocorticoid might be area-selective. For example, hippocampus and prefrontal cortex. which are critical for mood regulation, are proved to be more sensitive to damage from hypercortisolism [90].

## 4. Neurogenesis Hypothesis

Among the various hypotheses of depression, neurogenesis hypothesis is quite new. However, it has become the most highlighted thesis these years and is now believed to be the main road to the remission of depression [91]. This hypothesis emphasizes the critical role of new neurons of hippocampus in the mood control and pharmaceutical effect of antidepressants, and it was supported by abundant correlative studies, which mainly demonstrated that patients or animal models with depression had decreased neurogenesis and hippocampal volume, whereas antidepressants could enhance the neurogenesis of hippocampus [91].

Neurogenesis might also be important for PSD. Wang et al. [92] demonstrated that in the rat model of PSD, the proliferation and survival/neurogenic rates were reduced compared to the nondepression rats, and this reduction could be reversed by citalopram administration. Different theoretic evidence is in favor of neurogenesis in the pathogenesis of PSD. It is widely accepted that stress, which is common prior and posterior to stroke and identified as an important psychological factor of PSD but was not discussed in this paper, could influence neurogenesis in different ways [93], such as through glucocorticoid which has been proved to be involved in the decrease of neurogenesis [86]. The cross-talk between neurogenesis and immune system might also be rich. As we mentioned above, hippocampus is vulnerable to cytokines. Accumulating evidence suggests that various cytokines are associated with reduced hippocampus neurogenesis through different signaling pathways [86, 94]. For example, Sequin et al. [95] demonstrated that systemic administration of TNF-*α* could reduce the cell proliferation and survival within hippocampus. Besides, neurotrophin such as Brain-derived neurotrophic factor (BDNF) might also mediate the relation between neurogenesis and PSD. BDNF is critical for the maintenance of neuronal functions, plasticity, and regulation of neurogenesis which is important for mood control [96]. Several studies have proved that low BDNF level is also associated with PSD [97, 98]. It is reasonable to speculate that low BNDF level results in the decreased neurogenesis of hippocampus, thus leading to PSD. However, it is still unclear about the factors that determine the level of BNDF after stroke. Hypercortisolism and inflammation might partly account for the decreased BDNF after stroke with specific unclear mechanism [99, 100]. In the future, there are still a lot of studies required in order to understand the cross-talk among immune system, inflammation, BDNF, and neurogenesis.

## 5. Conclusion

The neurobiological pathogenesis of PSD is far from clear. Studies of PSD have provided various results about the different neurobiological hypotheses of PSD involving lesion characteristics, neurotransmitters, inflammatory factors, hormones, and neurotrophins. However, none of the multiple hypotheses is dominant enough to be identified as the only or main neurobiological pathogenesis of PSD. On the contrary, it is probable that PSD is caused by different biological factors which could coexist and interact in a complex way. In addition, PSD could be classified as different subtypes according to symptoms or time of onset. There were already some studies which indicated that the specific pathogenesis might be different for different subtypes of PSD [101]. Now studies about brain function network and neurogenesis are highlighted and some of them were proved to be significant for the understanding of PSD. In the future, in order to decrease the prevalence of PSD and improve the clinical outcome of stroke, more relevant studies are still required to explore the origin and clarify the process of PSD.
